# The effects of acute and elective cardiac surgery on the anxiety traits of patients with Marfan syndrome

**DOI:** 10.1186/s12888-017-1417-9

**Published:** 2017-07-17

**Authors:** Kálmán Benke, Bence Ágg, Miklós Pólos, Alex Ali Sayour, Tamás Radovits, Elektra Bartha, Péter Nagy, Balázs Rákóczi, Ákos Koller, Viola Szokolai, Julianna Hedberg, Béla Merkely, Zsolt B. Nagy, Zoltán Szabolcs

**Affiliations:** 1 Heart and Vascular Center, Semmelweis University, Városmajor Street 68, Budapest, 1122 Hungary; 2 Hungarian Marfan Foundation, Városmajor Street 68, Budapest, 1122 Hungary; 3 Department of Pharmacology and Pharmacotherapy, Semmelweis University, 1089 Nagyvárad tér 4, Budapest, Hungary; 4 GenePointPlus Hungary Ltd., János Zsigmond Street 7B, Budapest, 1121 Hungary; 5 Central European University, Nádor Street 9, Budapest, 1051 Hungary; 6 Institute of Natural Sciences, University of Physical Education, Alkotás Street 44, Budapest, 1123 Hungary

**Keywords:** Marfan syndrome, Cardiac surgery, Anxiety, Depression, Questionnaire

## Abstract

**Background:**

Marfan syndrome is a genetic disease, presenting with dysfunction of connective tissues leading to lesions in the cardiovascular and skeletal muscle system. Within these symptoms, the most typical is weakness of the connective tissue in the aorta, manifesting as aortic dilatation (aneurysm). This could, in turn, become annuloaortic ectasia, or life-threatening dissection. As a result, life-saving and preventative cardiac surgical interventions are frequent among Marfan syndrome patients. Aortic aneurysm could turn into annuloaortic ectasia or life-threatening dissection, thus life-saving and preventive cardiac surgical interventions are frequent among patients with Marfan syndrome. We hypothesized that patients with Marfan syndrome have different level of anxiety, depression and satisfaction with life compared to that of the non-clinical patient population.

**Methods:**

Patients diagnosed with Marfan syndrome were divided into 3 groups: those scheduled for prophylactic surgery, those needing acute surgery, and those without need for surgery (*n* = 9, 19, 17, respectively). To examine the psychological features of the patients, Spielberger’s anxiety (STAI) test, Beck’s Depression questionnaire (BDI), the Berne Questionnaire of Subjective Well-being, and the Satisfaction with Life scale were applied.

**Results:**

A significant difference was found in trait anxiety between healthy individuals and patients with Marfan syndrome after acute life-saving surgery (*p* < 0.01). The mean score of Marfan syndrome patients was 48.56 (standard deviation (SD): 5.8) as compared to the STAI population mean score of 43.72 (SD: 8.53). No difference was found between groups on the BDI (*p* > 0.1). Finally, a significant, medium size effect was found between patient groups on the Joy in Living scale (F (2.39) = 3.51, *p* = 0.040, η^2^ = 0.15).

**Conclusions:**

Involving psychiatric and mental-health care, in addition to existing surgical treatment interventions, is essential for more successful recovery of patients with Marfan syndrome.

## Background

Marfan syndrome is a rare, autosomal dominant genetic disease with the prevalence of 1:3000–1:5000, affecting connective tissue [1]. The life expectancy and quality of life of patients with Marfan syndrome are mainly determined by the presence and severity of cardiovascular lesions, such as annuloaortic ectasia, aneurysm and dissection [2, 3]. Marfan syndrome implies vascular events [4], which can be life-threatening and thus result in permanent anxiety for the patients. According to the 2012 ESC/EACTS guidelines [5], depending on the diameter of the aorta, prophylactic aortic-root replacement surgery is strongly recommended for the prevention of aortic dissection and rupture in patients diagnosed with Marfan syndrome.

There are several publications regarding aortic-root reconstruction surgeries carried out in Marfan patients. Gott and colleagues [6] reported on surgery of 675 patients (30% female, 70% male) with Marfan syndrome. In this group, 30% of the patients had had earlier aortic dissection. Shimizu and colleagues [7] performed 55 cardiac surgeries between 1987 and 2010 (34% female, 66% male), from which 50% had previous aortic dissection. In 2003 Yetman and colleagues [8] examined 70 Marfan patients (49% female, 51% male). Forty-nine percent of them had family history with the disease, and sudden cardiac death occurred among 27% of the patients. In this group 8% of the patients had previous surgery.

There are only few studies in the literature regarding the psychological and psychosocial factors of patients with Marfan syndrome.

In Hungary, there are approximately 2500 patients whose treatment and environment are likely to be different from those in the above mentioned studies. Because life-saving aortic surgery itself bears great physical and mental shock and trauma for the patients, we hypothesized that the level of anxiety and depressive mood is higher in Marfan patients than in the normal patient population. Furthermore we hypothesized that patients receiving preventive surgery are more satisfied with their lives due to their beliefs that their chance for aortic dissection or rupture is mediated and their chance for survival is therefore greater. Thus the aim of the present study was to investigate these issues in a population of Hungarian Marfan patients using tests similar to those carried out by Van Tongerloo and De Paepe [9] regarding differences in the levels of anxiety, depression and life satisfaction among Marfan patients with and without cardiac surgeries and compared their results to those from normal patient population [10].

## Methods

### Patients’ characteristics

All 45 patients (26 women and 19 men) involved in the study were diagnosed with Marfan syndrome utilizing the revised Ghent nosology [11], and had been enrolled in the National Marfan Registry (established and supervised by the Hungarian Marfan Foundation).

Based on the revised Ghent nosology the diagnosis of Marfan syndrome were established in case of each patient by systematically assessing the presence or absence of aortic involvement (either aortic dilation or dissection), ectopia lentis, positive family history, pathogenic sequence variations in the relevant genes and systemic involvement. By considering cardiovascular, skeletal, dural, eye and skin manifestations of Marfan syndrome specified by the nosology, a systemic score was calculated with a theoretical maximum of 20 points. Also according to the Ghent nosology systemic involvement was declared if the systemic score was greater than or equal to 7 points. Measured and calculated anthropometric parameters and the presence or absence of manifestations considered when calculating the systemic score are listed in Table 1 for each patient group.

**Table 1:** Clinical data

	1. Aortic dissection	2. Annuloaortic ectasia	3. Prophylactic	4. Non-operated
Patients	10	9	9	17
Male	4	6	6	3
Anthropometric (measured)				
Height	180.1 ± 10.1	183.9 ± 13.2	186.4 ± 13.7	180.2 ± 9.2
Lower segment (cm)	95.8 ± 8.6	100 ± 11.8	99.1 ± 6.8	94.3 ± 5.9
Arm span (cm)	190.3 ± 11.6	186 ± 17	192.6 ± 11.4	184.8 ± 7.5
Footsize	42.9 ± 2.2	43 ± 3.3	44.2 ± 4.1	42.2 ± 2.5
Weight (kg)	78.3 ± 13.4	71.9 ± 18.6	75.1 ± 19.3	69.6 ± 13.4
Anthropometric (calculated)				
Upper segment (cm)	84.3 ± 8.3	83.9 ± 9.2	87.3 ± 9.2	85.6 ± 6.8
Body Mass Index (BMI; kg/m2)	24.2 ± 3.7	21 ± 4.2	21.5 ± 4.5	21.3 ± 3
Body surface area (m2)	1.97 ± 0.2	1.91 ± 0.3	1.96 ± 0.3	1.86 ± 0.2
Upper segment - Lower segment ratio (USLS)	0.89 ± 0.12	0.85 ± 0.14	0.88 ± 0.09	0.91 ± 0.09
Arm span - Height ratio (ASHR)	1.06 ± 0.04	1.01 ± 0.04	1.04 ± 0.04	1.03 ± 0.03
Ghent nosology (%)				
Mitral valve prolapse	60	78	80	75
Pectus carinatum	40	44	80	63
Pectus excavatum requiring surgery	10	11	0	0
Reduced upper to lower segment ratio	40	44	20	19
Increased arm span to height ratio	70	11	10	19
Wrist sign	90	67	80	81
Thumb sign	80	67	100	94
Scoliosis of >20° or spondylolisthesis	80	56	90	94
Severe scoliosis	60	44	50	44
Reduced extension at the elbows	20	0	0	13
Medial displacement of the medial malleolus causing pes planus	30	22	70	69
Heel deformity	0	11	10	13
Pectus excavatum of moderate severity	20	22	40	31
Asymetric chest	40	22	70	44
Joint hypermobility	70	33	60	63
Highly arched palate with crowding of teeth	60	33	70	56
Facial appearance	50	22	60	38
Ectopia lentis	30	11	30	31
Myopia over 3 diopters	10	11	50	44
Spontaneous pneumothorax	0	11	0	13
Striae atrophicae (stretch marks)	100	44	70	56

Patients were assigned to surgical intervention groups following ESC/EACTS guidelines: Aortic dissection group, annuloaortic ectasia group, prophylactic group, and non-operated group. Participating patients completed the psychological questionnaires after medical consultation at the Heart and Vascular Center of Semmelweis University (Budapest, Hungary) in 2011 and 2012, with help of the medical staff. The research was approved by the Health Scientific Council, Scientific Research and Ethical Committee of Hungary. The study had ethical permission (13699–1/2011-EKU). Prior to the investigation all patients understood and gave informed consent. Patients in the study were volunteers; they were aware that they could withdraw from the research at any point without negative effects on their medical treatment. No patients withdrew during the course of the study (all completed).

### Descriptives

Marfan patients (*n* = 45) have filled the questionnaires: 26 women (58%, 38.9 ± 13 years old) and 19 men (42%, 38.9 ± 13 years old). Three sample groups were established based on the surgical procedure: group 1 had acute aortic surgery; group 2 had prophylactic surgery; group 3 had no surgery (see Table 2 for details).

**Table 2:** Types of intervention in Marfan patients

Name of the sample group	Cardiovascular diagnosis	Number of the patients (female/male)	Percentage of the patients
Acute aortic surgery	Aortic dissection	10 (6/4)	22% (10/45)
	Annuloaortic ectasia	9 (3/6)	20% (9/45)
Preventive prophylactic surgery	Prophylactic aortic-root reconstruction	9 (3/6)	20% (9/45)
No surgery	---------	17 (14/3)	38% (17/45)

### Applied psychological tests

In the present study Spielberger’s anxiety test (STAI) [12], the short form (9 items) of Beck’s Depression questionnaire [13], Berne questionnaire of subjective well-being [14] and the Hungarian version of Satisfaction with Life Scale (SWLS-H) were used [12]. STAI measures anxiety on a Likert-scale, and distinguishes trait anxiety (A-Trait), which defines one’s predisposition for anxiety, from state anxiety (A-State) which is the measure of current anxiety and the result of actual processed stimuli [10]. Due to ease of application, Beck’s Depression Questionnaire is used frequently in medical practice. It also utilises a Likert-scale, and was first applied to the Hungarian population by Rózsa and colleagues [15]. The Berne questionnaire of subjective well-being has several subscales: Personal problems, Somatic symptoms, Joy in Living, Depressive mood, Positive self-evaluation, and Positive attitude for life [16]. It was adapted for use in Hungary by Sallay [14]. The Hungarian version of Satisfaction with Life Scale (SWLS-H) consists of 5 items, uses a 7-level Likert-scale and measures global satisfaction with life, ignoring such linking factors like solitude and positive affectivity [17]. We used data from normal patient population in Hungary, derived from the publications mentioned above, as control for comparison.

### Statistical analysis of data

Mean, standard deviation (SD) and significance (*p*) values for MANOVA probes or Student’s t-tests were calculated with IBM SPSS Statistics for Windows, Version 22.0. Armonk, NY.

## Results

Due to the many combinations of the applied psychological tests, sample groups and the executed statistical analyzes, only the significant data are expounded below.

**Fig. 1:**
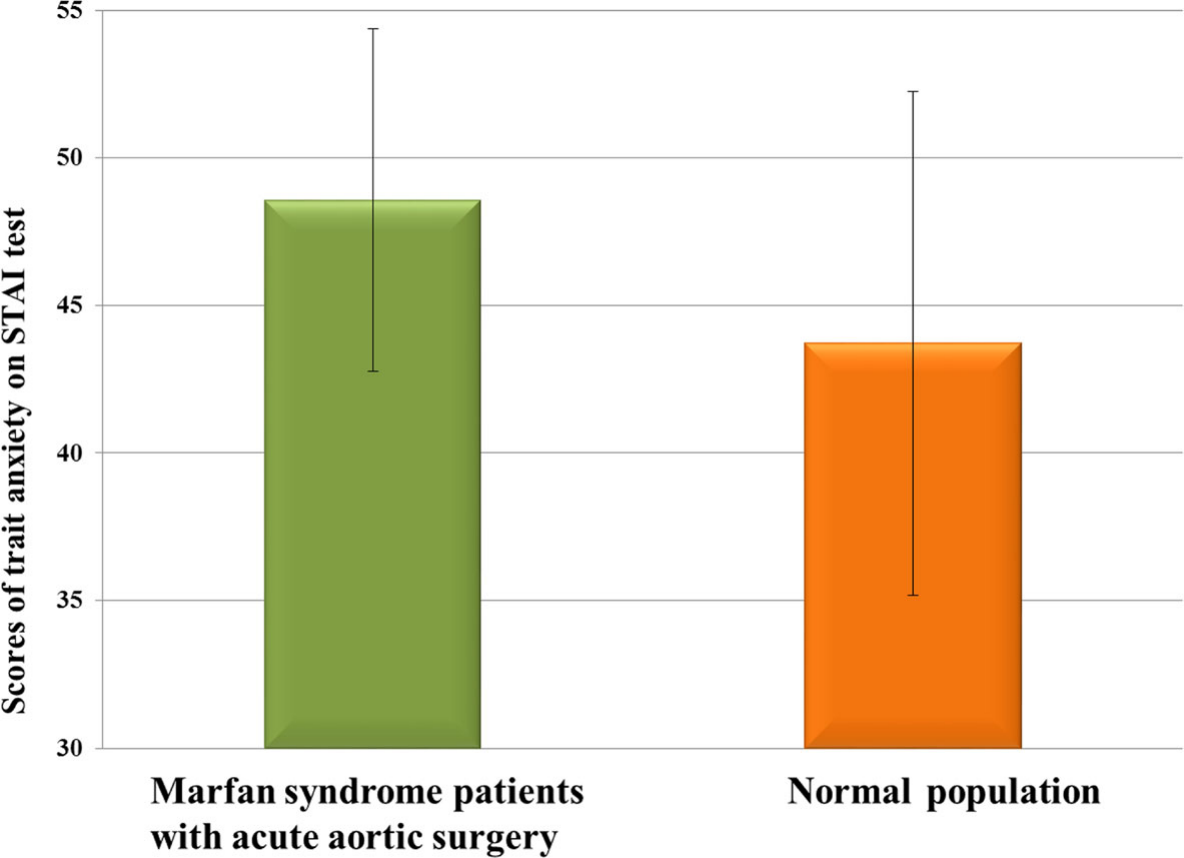
The average STAI (Spielberger’s anxiety) scores of normal patient population and Marfan syndrome patients with acute aortic surgery. The scores of trait anxiety - which determines the predisposition for anxiety - of this group of Marfan syndrome (*n* = 10) patients, are significantly (*p* < .01) higher, than the scores of the normal patient population (*n* = 376. Source: [10])

Using an independent samples t test, a significant difference was found on scores of trait anxiety between the Marfan population and the normal patient population (t(47) = 2.82, *p* < .01, Cohen’s d = .45). Average rates of high sample number tests of the Beck’s Depression Questionnaire [15] and STAI test [10], which were treated as the mean based on the mentioned source studies, were compared to the scores of Marfan syndrome patients with different surgical indications. The STAI trait anxiety scores of Marfan patients with life-saving surgery were significantly higher (*p* < 0.01) (Fig. 1). The average mean of Marfan syndrome patients was 48.56 (standard deviation: 5.8); STAI population mean score: 43.72, (standard deviation: 8.53; Sipos et al., 1978). There was no difference detected between Marfan and control for the BDI (*p* > 0.1).

A further question raised in the research was whether any difference may exist between the patient groups on the scales used. A multivariate analysis of variance (MANOVA) was applied on scale and subscale scores as dependent variables, and with Patient Group as a between-subject factor (three levels: No surgery, Prophylactic and Acute aortic surgery). A significant, medium size effect of patient groups on the Joy in Living scale (F(2,39) = 3.51, *p* = 0.040, η^2^ = 0.15) was found. Pairwise comparisons revealed that patients with life-saving surgery (factor level Acute, M_Acute_ = 17.22, SD_Acute_ = 3.59) scored significantly higher on the scale than patients with no surgery (M_Nosurg_ = 14.00, SD_Nosurg_ = 3.44). There was no other effect of Patient Group in the MANOVA (all other Fs < 1.42).

Nevertheless, in other aspects of our study, significant statistical differences between the groups were not indeed expected, due to the small number of the patients involved in the recent study and many factors, which influence the applied tests (e.g. time of the diagnosis, how the patient is aware of the health condition, which other illnesses the patients have, etc.).

## Discussion

For most people, life-saving aortic surgery is a traumatic event, whereby they could experience the vulnerability of their lives, when they have to live in a constant, life-threatening and hardly definable distress. It can be a significant factor contributing to a permanent anxiety state, which is normally not present in healthy individuals without Marfan syndrome.

Based on the statistical analysis it can be concluded that patients suffering from Marfan syndrome and required life-saving aortic surgery due to aortic dissection experienced a greater anxiety in everyday life. This effect was unlikely to be a consequence of stress associated with cardiac surgery, as there was no significant difference between patients with prophylactic aortic surgery relative to the normal patient population.

Fusar-Poli and colleagues [18] examined 36 Marfan syndrome patients and found that the disease had a negative effect on the quality of life, was associated with increased psychological stress, and increased patient risk for the occurrence of certain psychiatric diseases. Hofman and colleagues [19] investigated nervous system and cognitive development of 30 children with Marfan syndrome. Based on their results, 50% of the children showed one or more neuropsychological deficits and symptoms similar to depression, which could be related to the serious motoric incoordination associated with Marfan syndrome. Baeza-Velasco and colleagues [20] showed that certain diseases of connective tissue (e.g. Marfan and Ehlers-Danlos syndrome) may be associated with various psychiatric symptoms, such as those of anxiety and depression.

Connective tissue disorders are a source of frustration for patients. Marfan syndrome may contribute to a sense of loss of personal efficacy due to physical disability and the need for cardiovascular intervention [21]. Furthermore, this syndrome has an autosomal dominant heritability, which results in a 50% chance of transmitting the mutant FBN1 gene to offspring; this knowledge may further augment persistent anxiety [22] about pregnancy for both patient’s own health and health of their children [23]. After diagnosis, having a lifelong, potentially disabling disease with the potential for multiple organ system involvement may intensify challenges in daily life, contributing to decreased quality of life and greater psychological distress [23]. Velvin and colleagues [23] noted that further research is required in order to better understand the potential importance of the psychosocial aspects of Marfan syndrome, as cardiac surgical intervention affects the whole life of the patient and might influence the psychological well-being of these patients as has been described here. It is necessary to inform the MFS patients about their condition after clinical diagnosis, and including consultation with a psychologist might help in processing this information in a healthier fashion. Finally, this additional support could foster better compliance and better treatment outcomes.

Our study highlights the presence of high anxiety in Marfan syndrome patients who have undergone life-saving aortic surgery, which is important for healthcare experts to note. Also deserving further consideration, in line with the findings of the research literature is that continuous anxiety has many negative effects on mental and physical health of patients [24, 25].

## Conclusions

**Fig. 2:**
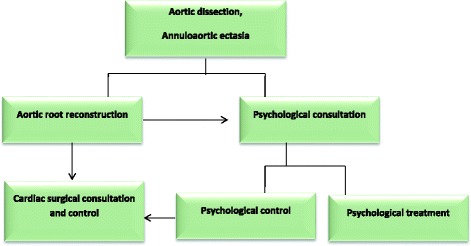
Complex program of cardiac and psychological examination, consultation and treatment of Marfan syndrome patients**.** After an aortic-root reconstruction surgery, psychological consultation is recommended alongside cardiac surgical control, and if necessary, psychological treatment. The psychological consultation and control is justified by the high anxiety level experienced in Marfan syndrome patients after life-saving aortic surgery as showed in this study

It would be reasonable to create a comprehensive psychological healthcare program to help Marfan patients to cope with anxiety. Since life-saving cardiac surgeries themselves are serious stress factors it is important to involve psychiatric and mental-health professionals at those institutions which perform cardiac surgery on Marfan patients [26]. Thus, both cardiovascular and psychological controls are significant in order to purposefully and effectively improve the quality of life of Marfan syndrome patients (Fig. 2). Further investigations are necessary to clearly understand the correlations revealed in this research.

## Abbreviations

BDI: Beck Depression Inventory
ESC/EACTS: European Society of Cardiology/European Association of Cardio-Thoracic Surgery
MANOVA: Multivariate Analysis of Variance
SD: Standard Deviation
STAI: State-Trait Anxiety Inventory
SWLS-H: Satisfaction With Life Scale-Hungarian version
